# Uterine Cancer

**Published:** 1894-12

**Authors:** George R. West

**Affiliations:** Chattanooga, Tenn.


### UTERINE CANCER.*

By GEORGE R. WEST, B.S., M.D.,

CHATTANOOGA, TENN.

I offer no apology for consuming a portion of the time of this association by the presentation of so trite a subject as Uterine Cancer. I know that the pages of the journals and the time of the medical societies and the best work of some medical lives have been devoted to this topic, but has not their failure of successfully mastering the subject rather made it more obligatory upon us to spend more time in research than to cast it aside as a subject of which we have heard enough?

A disease which has existed since the memory of man, and which kills five and five-tenths per cent. of our women, should have the constant study, not alone of the gynecological specialists, but every practitioner has a responsibility in these statistics.

I claim, and hope to show you, that upon the family physician rests the responsibility of reducing this mortality.

The consensus of opinion among modern medical men is that cancer is, primarily, a local disease, and that the systemic contamination is secondary. It is apparent here, as elsewhere, that the pathological condition must be the basis on which rational treatment can be formulated. Perhaps the accepted doctrine of the pathology of malignant disease is the presence of cells normal to the body, but out of time and place. What we call carcinoma is recognized to be an atypical epithelial formation.

Thomas tells us that the cancer is three times more frequent in women than in men, and also three times more frequently met with in the uterus than in other organs.

He also states that the decades in a woman's life, as regards its frequency, are in the following order, viz.: 40–50, 30–40, 20–30, and 50–60, and that few occur before 20, or after 60.

The parasitic origin of cancer has received much study. Mr. Clark, of London, has this year announced the discovery of the

*Read before the Tri-State Medical Association, October, 1894.

true parasite, but a careful examination of his claim proves it unfounded. Many conflicting theories will have to be harmonized before we reach a conclusion on this important theory. At present no facts, historical or otherwise, compel the assumption of a parasitic origin of carcinoma, while there are very strong and valid arguments against such assumption.

A factor in the etiology mentioned by Skene, Emmet, and Thomas is that cancer of the uterus is in proportion to the frequency of pregnancies. Emmet gives statistics in which sixty per cent. of the cases have the disease confined to the cervix at the beginning.

Dr. Wm. Goodell says that in a large proportion of the cases the disease begins in the vaginal portion of the cervix. It does this because this part of the womb bears the brunt of the injuries sustained in coition and parturition.

The cancerous nodule or ulcer starts usually in the notch of a torn cervix, and it is, therefore, most commonly found in women who have borne children. Corroboration of this opinion is the fact observed by many that cancer of the cervix is rare in virgins, but that cancer of the body of the uterus is a disease of spinsters and sterile wives.

Believing, as he does, that cervical lacerations may produce cancer, Howard Kelly lays down the following rules for the obstetrician, which, if carried out, would very much increase his labors:

1st. Examine each patient two or three months after confinement and carefully note the condition of the cervix and uterus.

2d. Carefully describe position and depth of cervical tears and appearance of lips.

3d. Patients past thirty-five in whom the cervical lips do not appear perfectly sound should be kept under observation and examined at intervals of from six to eight months.

4th. Every woman over thirty-five, with a cervical tear, should be examined at least once a year for ten years, or longer, if the appearance of the lacerated area is not perfectly healthy.

5th. A suspicion of inheritance would give these rules peculiar force. If laceration of the cervix is the direct cause of cancer of the cervix, we must have some predisposing cause, as the number of cervical tears met with in practice are out of all proportion to the number of cases of malignant diseases of that organ. This predisposing cause we do not know.

The study and research by pathologists and bacteriologists have taught us nothing by which we can explain the origin of cancer. The origin being unknown, we are deprived of the important key to its successful treatment. Having such a vague idea of the elements producing this dreadful malady, we are, nevertheless, only too familiar with the symptoms which we must treat.

The ordinary symptoms of carcinoma in the usual order of their appearance are: Pelvic pain, ichorous leucorrhea, hemorrhages, fetid discharges, and general cachexia.

It is well known these make up an advanced case of uterine cancer, but that many or all of them may be absent in any given case.

These symptoms being so common, I take it all are familiar with them and I will not waste time elaborating them.

We are especially interested in the first symptoms of cancer as it is to them, as warning us of a probable danger, we want to call your attention.

"The first symptom in five-sixths of all cases of cervical cancer," says Winckel, "is hemorrhage." But by this he does not mean the profuse loss of blood, such as sometimes occurs during the stage of ulceration. This hemorrhage is the result of local hyperemia, and has different features according to the age of the patient.

Coe wishes to lay particular importance to the symptom of slight hemorrhage after coitus. If you take the trouble to question patients about this sign, you will be surprised to see how often it can be identified as the initial objective symptom of malignant disease.

A digital examination is the only trustworthy way of arriving at a correct diagnosis, and I cannot urge too strongly the following advice from Hart and Barbour: "In all cases in which a patient over forty years of age seeks advice with symptoms referable to the pelvis, a careful examination should be made." If these symptoms be painful coition, with a bloody discharge afterwards, stubborn pelvic pain, free leucorrhea, and especially of irregular uterine hemorrhages, the finger will but verify the diagnosis.

Suppose the patient to have borne children and be approaching fifty and you have all the symptoms necessary. The belief is still prevalent that the menopause is a time when women may have hemorrhages that are physiological. How many patients I have seen who told me that their pain, or increased or persistent hemorrhage, had been explained to them as the "change of life."

I cannot be too strong in my denunciation of this false belief. That menopause which is accompanied by irregular symptoms is one to cause alarm, and will call for careful investigation by the attendant physician.

It is well to keep in mind that any woman may have cancer, and in thus looking for the worst we cannot be sorely disappointed.

The result of a digital examination is not always positive. The finger gives more information than the speculum.

The examination will disclose a large indurated cervix, presenting a unilateral (or, rather, more commonly, a bilateral) laceration, with eversion of the lips. The soft velvety sensation imparted by the erosion may be masked by the general induration, which, it is important to note, is not confined to the angle of the tear. The everted mucous surface bleeds more readily than a simple erosion. Instead of the ovula nabothi usually felt, small hard nodules are often present. At this stage the uterus is movable. In later stages the enlarged cervix, ragged and friable edges, which easily bleed, make up the diagnosis.

If we still have doubt, the microscope is usually called upon to verify the suspicion. We are instructed to excise a wedge from the cervix and submit it to the pathologist, but even this examination may be negative, for it is not always easy to make out the nature of the trouble from the small wedge we are likely to furnish.

I have not been led to place much reliance upon the use of the microscope. If the symptoms do not warrant a diagnosis microscopically, the patient in private practice will seldom permit a serious operation when indicated by the microscope. The microscope is of greater value to the pathologist than to the surgeon, and hence it teaches us what microscopic systems are of value.

I am sorry to say, in connection with the treatment of this malady no cure has ever been discovered. There must, however, be a cure somewhere, as I do not believe a merciful Creator would afflict mankind with so dreadful a disease without furnishing a relief. Many noble men have spent the greater part of their lives in research, and still the secret is hidden.

The aniline dyes have not proven themselves worthy of the name, though much benefit is ascribed to their use. Recent investigations and experiments with the germs of erysipelas in the cure of cancer are quite favorable, but it is too early to say positively we have in them a cure. Electricity can only be palliative. In the absence, then, of a reliable cure, and believing, as most of us do, that cancer is at first a local disease, we are forced to accept its removal as the best treatment. Statistics show us a proportion of complete immunity by operation on cancer of the uterus, of thirty-five to forty-four per cent. (Curtis.)

The first step towards the improvement of these results must come through the family physician—the general practitioner. Not until he is fully convinced that operation will cure the disease if attacked in the early stages, and not until he is able to make an early diagnosis, can we secure the full value of this method of treatment. On him depends the entire question.

The variety of operation selected depends upon the surgeon and the patient. It depends upon the surgeon because, as a rule, the men who do this work are wedded to one operation and insist upon that in all cases. It depends upon the patient, because we must always consider their condition as to whether their general health will admit of operating at all.

As I view it, a doctor who takes a case of uterine cancer for treatment should stand in a neutral position as far as a selection of a method of operating, and should do what the condition of the patient demands. If the cancer has only affected the cervix, and you are reasonably certain of that, a high amputation of the cervix by the knife, or actual cautery, would be advisable because of less risk; but if the disease is corporeal, or has extended into the body from the cervix, the method would be total extirpation.

As it is impossible to know how far up a cervical cancer has extended at the time of operation, I hold that the method promising the greatest number of good results is vaginal hysterectomy.

As operators become more familiar with the technique of this method, the statistics improve in their mortality rate, so that I predict for it, when the operation shall be done early enough, the same brilliant results as those attained in abdominal section.

The greatest hindrance toward perfecting these statistics, as I have before said, is the fact of the surgeon not seeing the patient early enough. The majority of uterine cancer cases that have come to me for treatment have been past the time when they could be benefited by operative interference, and this seems to be the experience of all operators. If the neoplasm has invaded the tissues around the uterus, and it is not probable we could remove the organ and leave healthy structures behind, we had better not resort to operative interference at all. Though the movability of the uterus is considered the crucial test, yet the fact of its being fixed does not always contraindicate an operation, for it may be immovable from inflammatory products.

But, what shall be done with these cases where operation is not advisable? Shall they be dismissed with a statement of their condition and advised to incur the opium habit? By all means, *no*. We can benefit such patients by hopeful words and giving them something to do. We thus make a great improvement by treating the mind. Give them washes to act as deodorants; give local injections of salicylic acid or of pure alcohol, or prescribe an opiate in a concealed form, to relieve the pain.

The curette is used as a palliative in advanced cases. By it we remove necrotic tissue, and thus control hemorrhage and fetor. The action of the curette can be supplemented by following it with the use of chemical caustics or the actual cautery. Plasters, paste, etc., cannot be used without poisoning the patient, and some form of the actual cautery is the only proper escharotic which should be deemed advisable.

Dr. J. W. Hulke, one of the surgeons to Middlesex Hospital, which has had a regulation requiring the surgeons in charge of patients in cancer wards, to try every alleged remedy, the composition of which is not "secret," and for which, obviously, there are no sufficient grounds for judging that its effect, if any, will be injurious, writes the following to the *London Lancet:*

"It has fallen to me to make trial of various remedial measures, suggested here and abroad, for the relief and cure of sufferers from cancer, in the course of some twenty-five years. Of such "pharmaker" I mention "Fell's Paste" (the active ingredient in which is zinc chloride, colored with red snake root), slow injection of acetic acid through a capillary tube, sundry escharotic pastes and powders having arsenic as their essential component, condurango, and more recently, chian turpentine. Also, besides these, the dietetic treatment laid down by Beneke, without and with association of arsenic administered internally, recommended by Esmarch. Several years ago the conjecture that cancer might have a parasitic origin, induced me to try cinnamon; being induced in the selection of this by the common ascription of germicidal properties to aromatic oils, and by my recollection that my old teacher, Dr. A. Farre, held strongly that cinnamon had something like a specific or particular influence over some uterine functions, a belief which led him to prescribe large doses of the tincture in certain cases of dysmenorrhea. I gave cinnamon a long trial, chiefly in cases of cancer of the uterus, but also in that of other organs. The result was wholly negative."

While, therefore, there are but few of the cases of cancer of the uterus as we now see them that offer a good prospect of relief from surgical interference, yet in many we can relieve the pain, retard the progress of the disease, and add months, if not years, to the life.

My remarks have purposely been superficial, with the idea of indicating the fact that our knowledge of uterine cancer is not exact. We have, then, a prevalent and fatal malady without exact knowledge. Is it not a fruitful field for investigation? I hope the day is not far distant when the world will be electrified by the discovery of the specific for cancer.

